# Comprehensive care and accompaniment: presuppositions, attitudes, and scope

**DOI:** 10.3389/fpsyg.2026.1722294

**Published:** 2026-03-04

**Authors:** Paula María Núñez-Sánchez, Carmen de la Calle Maldonado, Cecilia Castañera Ribé

**Affiliations:** 1 Instituto de Acompañamiento, Universidad Francisco de Vitoria, Madrid, Spain; 2 Escuela Internacional de Doctorado, Universidad Rey Juan Carlos, Madrid, Spain; 3 Departamento de Humanidades, Universidad Francisco de Vitoria, Madrid, Spain

**Keywords:** accompaniment, attitudes, caregiver, competencies, comprehensive care, culture of accompaniment, preventive care, vulnerability

## Abstract

This article proposes a theoretical-conceptual reflection on accompaniment as a fundamental relational style of comprehensive care, with a primary focus on the healthcare field. Based on a narrative and critical review of philosophical, ethical, and healthcare literature, the work draws on the anthropology of care—especially Francesc Torralba’s personalist philosophical perspective—to establish care as a structural dimension of the human condition, intrinsically linked to vulnerability and relationality. The hypothesis is that accompaniment constitutes a privileged pathway for the humanization of healthcare in contexts marked by increasing technification and standardization of care. From this perspective, accompaniment is understood as a comprehensive practice oriented toward the whole person, addressing the biological, psychological, social, and spiritual dimensions in an articulated manner. The analysis incorporates a multidimensional understanding of vulnerability—ontological, situational, relational, and technological—emphasizing its relevance in the post-COVID-19 context and its ethical implications for the clinical relationship and moral responsibility. The article concludes by highlighting the value of accompaniment as a relational framework for more humanized and person-centered healthcare practices.

## 1 Introduction

Care is a structural dimension of human experience and is intrinsically linked to its relational condition. Human beings are beings with others, open to others, who need to be cared for from the very beginning of their existence in order to develop fully. This condition of need and dependence is not an accidental deficit, but a constitutive feature of the person. In this sense, Torralba defines care as “a constitutive activity of the human being, because it understands man as a being who needs to be cared for in order to remain what he is; as a being who can only develop his potential if he is cared for” (2005).

From this anthropological perspective, vulnerability is not conceived as mere fragility or deficiency, but as a fundamental condition that makes ethical responsibility and the relationship of care possible. Far from nullifying personal agency, vulnerability constitutes the space from which the need—and the possibility—of being accompanied and cared for emerges, especially in contexts of illness, suffering, or dependence. Recent literature emphasizes that this relational understanding of vulnerability has taken on renewed centrality in the healthcare field, particularly in the wake of the COVID-19 pandemic, by highlighting human interdependence and the limits of overly technified or depersonalized models of care (Johansson and Wickström, 2023).

This article proposes a theoretical-conceptual reflection on the person as a being constitutively oriented toward care, with a primary focus on healthcare. Based on various theories of care developed over the last century (Heidegger, 1927; Lévinas, 1961; Gilligan, 1982; Noddings, 1984; Torralba, 1998; Torralba, 2000; Torralba, 2005; Boff, 2013; Cortina, 2013; Toro, 2018), the anthropological assumptions that underpin the care relationship are addressed, and the hypothesis is put forward that accompaniment constitutes the privileged relational style for enabling comprehensive and humanized care.

In this framework, accompaniment is understood as a relational practice that favor’s active presence, listening, co-responsibility, and recognition of the dignity of the vulnerable person in their bio-psycho-socio-spiritual condition. From this perspective, comprehensive care is not limited to attending to physical or technical needs but involves a personal and ethical response to the other’s vulnerability. This understanding is particularly relevant in the contemporary healthcare context, characterized by a growing tension between technological efficiency and the need to humanize healthcare practice.

The aim of this work is therefore to clarify the concept of accompaniment from an anthropology of care, to identify the fundamental attitudes and skills required for comprehensive care in the face of human vulnerability, and to reflect on the scope of a culture of accompaniment as a means of humanizing healthcare, promoting health and preventive care.

This work adopts a theoretical-conceptual approach, grounded in a narrative and critical review of philosophical, ethical, and healthcare literature on care, accompaniment, and vulnerability. The selection of authors and sources has been guided by their relevance to the development of care theory, their impact on contemporary debates on humanization and person-centered care, and their consistency with a personalist anthropological framework. The aim is not to provide an exhaustive review but rather to offer a reasoned conceptual articulation that clarifies accompaniment as a category of comprehensive care.

## 2 Comprehensive care and accompaniment

### 2.1 The concept of accompaniment

Strictly speaking, accompaniment is understood as a type of interpersonal relationship structured around three key ideas: “being with someone,” “being on a journey toward,” and participating in a “dynamic of collaboration and exchange” (Simard, 2016). It is therefore a cross-cutting practice that spans different disciplines, professions, and life experiences.

From an etymological point of view, the term derives from the Medieval Latin cum-panis, evoking the image of life as a journey and of human relationships as companionship among pilgrims who share their efforts and food (Cencini, 2008). This etymological root implies the possibility of meaningful interaction, highlighting the reciprocal and enriching nature of the accompaniment relationship.

Based on this tradition and recognizing the person as a being in need of care, it is appropriate to propose a definition of accompaniment with several meanings. Firstly, it is a relational style oriented toward human growth in all stages of life (Barahona et al., 2002). Secondly, it constitutes a dynamic process, a path of personal and community encounters and disagreements that point to integral development and vocational fulfillment (González Iglesias, 2023). Finally, accompaniment can be understood as a response to an anthropological need: companionship as an intrinsic condition of human beings, tailored to their needs (González Iglesias and la Calle Maldonado, 2020).

As accompaniment is a relational practice present in various areas of human life—educational, family, or community—this reflection focuses primarily on healthcare. The vulnerable person referred to in this study is understood as someone who, in situations of illness, suffering, dependence, or fragility, requires a caring relationship that recognizes their dignity, uniqueness, and bio-psycho-socio-spiritual condition. From this perspective, the humanization of care refers to approaches that counteract the risks of depersonalization associated with excessive technification and standardization in healthcare, emphasizing relational presence and ethical responsibility.

The growing prominence of accompaniment in contemporary societies can be understood as the expression of profound cultural transformations that have placed the person at the center of social life and care systems, particularly in the healthcare field. This shift represents a paradigm shift from care models focused predominantly on disease, technical efficiency, or the standardization of processes, and directs attention toward a relational and ethical understanding of care, in which the uniqueness of the individual takes center stage.

From an anthropological perspective, this reconfiguration finds a solid foundation in the conception of care as a constitutive dimension of human existence. Torralba argues that human beings are essentially vulnerable beings who need to be cared for in order to become fully who they are, so that care is not an ancillary activity but a necessary condition for the development of their potential and the preservation of their dignity (Torralba, 2005). This anthropological understanding allows us to interpret accompaniment not only as an organizational or relational strategy, but as a practice that responds to an ontological need of human beings.

In line with this perspective, recent international literature points out that the centrality of the person has become a structuring principle of care policies and practices. Winance and Bertrand (2024) describe how, since the 1980s, *personalization* has come to occupy a central place in the evolution of welfare and healthcare systems, understood as the adaptation of care to the specificities of each person through the development of person-centered care. This shift means that healthcare interventions are organized around individual life trajectories, needs, and values, reinforcing continuous support practices tailored to the uniqueness of those receiving care.

In healthcare, person-centered care has established itself as an international paradigm for quality care. This approach recognizes people as active participants in the care process, bearers of experience, values, and decision-making abilities that must be integrated into clinical practice (McCormack and McCance, 2021; Santana et al., 2018). From this perspective, support takes on a relational and longitudinal character, transcending the specific clinical act and focusing on the comprehensive support of the person throughout their health-illness process.

Recent studies also emphasize that this movement toward person-centered healthcare has a global and cross-cutting scope. Tyagi et al. (2025) point out that consolidating this paradigm requires genuine recognition of the *personhood* of all those involved in care, thereby reinforcing its ethical and relational dimensions. This orientation aligns with the anthropology of care proposed by Torralba, in that both perspectives conceive the person as an end in themselves and care as a practice that recognizes and responds to their constitutive vulnerability.

Finally, the convergence between person-centered and accompaniment is closely linked to contemporary discourses on the humanization of healthcare. The literature points out that the processes of technification and standardization have generated dynamics of healthcare depersonalization, in the face of which it is necessary to recover care practices based on empathy, presence, and recognition of human dignity.

From this perspective, the humanization of care is not conceived as an optional addition, but as the practical and anthropological expression of an anthropological understanding of human beings as vulnerable subjects who need to be accompanied in order to develop and sustain themselves in contexts of illness and suffering.

Together, Torralba’s anthropology of care and the recent international literature on person-centered care allow us to understand healthcare accompaniment as an ethically grounded and culturally situated practice that responds to both structural transformations in contemporary societies and to a deep understanding of the human condition. In this work, accompaniment is understood as integral insofar as it is oriented toward the person as a whole, attending to the biological, psychological, social, and spiritual dimensions in an articulated manner. This conception is based on Torralba’s aforementioned anthropology of care, for whom care constitutes a structural dimension of human existence and cannot be reduced to partial or exclusively technical interventions. This specific proposal of integral accompaniment is part of the tradition of philosophical personalism, insofar as it places the person at the center of ethical reflection and recognizes their irreducible dignity, relational nature, and constitutive vulnerability. This perspective is particularly relevant to a reflection on care and accompaniment, as it allows for the integration of freedom, dependence, and responsibility within the same anthropology. As a starting point, let us consider that care is a natural consequence of accompaniment when approached from this perspective.

### 2.2 Human vulnerability

Vulnerability is a constitutive feature of the human condition. Nussbaum (1995) emphasizes that it is an essential element of what it means to be a person. This need is manifested as a demand for physical, emotional, and spiritual support, highlighting the importance of care. Recognizing one’s own vulnerability and that of others opens up the possibility of establishing authentic and supportive relationships (Toro, 2018). Far from being conceived solely as weakness, vulnerability reveals the interdependence that underpins social life. From childhood, human beings depend on the care of others during a prolonged period of development, which creates lasting family and community bonds. Caring not only ensures survival but also strengthens social cohesion and encourages cooperation. Thus, care is a central element in building networks of trust and sustaining communities.

This process begins with recognizing fragility, which opens up spaces for trust and empathy. Caring means accepting others’ vulnerability and responding with attention and compassion. As Coca et al. (2022) point out, vulnerability and care are two sides of the same ontological reality, in which the balance between awareness of fragility and the ability to support each other is at stake.

In this vein, Cencini (2008) reminds us that accompaniment expresses the relational nature of human beings, as bonds capable of caring and, at the same time, in need of care.

The concept [of accompaniment] expresses the relational nature of human beings, and more specifically the quality of the bond that unites people, one responsible and capable of caring for the other, but also in need of their help and presence. At the same time, this concept refers to the classic idea of life as a journey and of human relationships as a companionship between pilgrims who share their efforts and the bread of the road. Finally, the practice of helping finds its interpretative parameters in psycho-pedagogical theories that favor a non-directive approach in the helping relationship (Cencini, 2008).

From this perspective, accompanying and caring appear as inseparable practices in the face of human vulnerability. This concept has acquired renewed centrality in bioethical reflection and health sciences in the wake of COVID-19, not only because of the exposure of individual fragilities, but also because of the visibility of relational, institutional, and socio-technical vulnerabilities that permeate health systems. Contemporary literature has questioned the simplified understanding of vulnerability as a fixed attribute of certain “vulnerable groups,” proposing more dynamic models that allow for the identification of layers or dimensions that are activated according to contexts and relationships. Along these lines, it has been argued that vulnerability should be approached as a multilevel phenomenon, sensitive to triggering conditions and *cascading effects*, thereby avoiding the homogenization of individuals or groups and enabling more precise interventions (Victor et al., 2022).

In the field of care, this perspective is particularly relevant because vulnerability not only affects those who receive care but also those who care for and accompany them. Recent studies have shown that the pandemic has helped bring the often invisible vulnerability of caregivers to the fore and have proposed a rethinking of care relationships to recognize how exposure to uncertainty, moral burden, and institutional constraints shapes specific vulnerabilities among care providers (Habran et al., 2024). This perspective allows us to understand vulnerability as a relational phenomenon, in which asymmetries of power, responsibility, and responsiveness decisively influence the experience of care and its humanization.

Furthermore, the rapid expansion of healthcare digitalization in the post-COVID period has intensified an emerging field: technological or socio-technical vulnerability. Empirical evidence shows that digitalization reconfigures care work, can reduce face-to-face encounters, and adds tensions related to work pace, communication, information security, and system vulnerabilities (Kaihlanen et al., 2023). At the same time, recent reviews of digital technologies in healthcare warn that their incorporation may shift and redistribute moral responsibilities among professionals, patients, and technology, creating “responsibility gaps” and new ethical risks, which require a rethinking of accountability and the protection of vulnerable people (Meier et al., 2025).

This scenario has also prompted research into the humanization of care amid automation and standardization, which can lead to depersonalization of care for patients and burnout among professionals. A recent systematic review identifies humanization actions in nursing care, as well as barriers and facilitating strategies, placing the relational dimension (presence, communication, recognition) at the core of counteracting dehumanizing trends (Reyes-Téllez et al., 2024). From the perspective of the ethics of care, it has also been emphasized that technologies such as AI (e.g., therapeutic *chatbots*) can affect human relationships and emotions, and that regulatory frameworks focused exclusively on *“responsible* AI” are insufficient if they do not explicitly incorporate vulnerability, dependence, duty of care, and power asymmetries (Tavory, 2024).

Taken together, the post-COVID literature allows us to propose an operational typology of vulnerability that is particularly useful for informing healthcare support: (a) ontological vulnerability, inherent to the human condition; (b) situational vulnerability, associated with illness, dependency, or disability; (c) relational vulnerability, derived from asymmetries in care relationships; and (d) technological vulnerability, intensified after the COVID-19 pandemic in relation to the processes of digitalization and the increasing use of technology in care (Johansson and Wickström, 2023) and their effects on the clinical relationship and moral responsibility. These dimensions are not mutually exclusive; they overlap and can intensify one another. Specifying them strengthens the argument that accompaniment (and its comprehensive orientation) constitutes an ethical and relational response to human fragility, especially in healthcare systems where increasing technification requires explicit humanization strategies. Recognizing these dimensions allows us to understand accompaniment as a relational and ethical response to human fragility.

### 2.3 The concept of care

From an anthropological perspective, care is a relational act that involves attention, responsibility, and responsiveness to others’ needs. It is not limited to the physical, but includes the emotional, social, and spiritual, constituting a pillar of social cohesion.

Its necessity arises from biological and social vulnerability present in childhood, illness, and old age, as well as from the emotional interdependence inherent in community life. Anthropologically, care strengthens bonds and generates reciprocity, contributing to both individual and collective well-being. In many cultures, care is conceived as a fundamental moral and relational duty that contributes to preserving community cohesion and sustaining social life (Boff, 2013; Rocchetta, 2016; Torralba, 1998; Torralba, 2005).

Care is not only essential for survival, but also for emotional, social and ethical development. Its relationship with accompaniment is evident at every stage of the life cycle and across various settings, such as the family, school, work, and culture. At the same time, the social organization of care can reflect inequalities and reproduce dynamics of exclusion.

Human beings, like social and relational creatures, have a profound need for care that manifests itself throughout their lives. As we have seen, dependence on the care of others is evident from birth, and this need evolves as we grow. Care is not only an indispensable condition for survival, but also a decisive factor in a person’s emotional, social and ethical development. From this perspective, the definition of accompaniment makes sense when linked to the different areas and stages of the life cycle. Human development, usually segmented into phases that respond to physical, emotional, and social transformations, requires specific forms of care and accompaniment for each moment. Likewise, the reference to areas integrates not only the evolutionary stages but also the relational and contextual spaces—such as family, educational, professional, and cultural life—recognizing that each contributes in a particular way to the integral development of the person in its various dimensions and capacities.

Various theories and thinkers have explored this concept in depth. From Heidegger’s existential philosophy to the ethical theories of Gilligan and Nodding’s, and beyond Torralba, care is presented as an essential aspect of human experience. These authors have contributed significantly to the understanding of care from various perspectives:

Martin Heidegger (1927), with his philosophy of “being-in-the-world,” provides a framework for understanding care as an integral part of human existence. For this author, care is an existential structure that reveals the intimate relationship between human beings and their world, where concern for others and the environment are essential to authenticity and the meaning of life.

Lévinas (1961), in his work “Totality and Infinity,” argues that the recognition of the other and their suffering is essential to our humanity. The inability to care for others can lead to the dehumanization of both the caregiver and the person cared for.

Carol Gilligan (1982) criticizes the traditional ethics that predominated in moral theory, focusing on justice and individual rights and generally reflecting a masculine perspective. Instead, she argues that women tend to focus on care and interpersonal relationships. Gilligan proposes that the morality of care should be recognized as equally important, highlighting how care involves responsibility and attention to the needs of others, promoting a more relational and contextual ethic.

Noddings (1984) develops an ethics of care as a fundamental way of understanding morality. She argues that care should be at the core of ethical and moral education, and that caring relationships are essential for human development. She emphasizes that care is not just an act, but an attitude that manifests itself in relationships, where empathy and mutual understanding are required. Her approach highlights the importance of personal bonds and of recognizing others’ vulnerability.

Leonardo Boff (2013) argues that care is an ethical imperative that should guide our actions. It is based on the principle that all human beings are interdependent and that the well-being of one is linked to the well-being of others. In his works, Boff links care to spirituality, emphasizing that caring is a way of loving and respecting life in all its forms and involves recognizing and attending to the needs of others, as well as establishing meaningful bonds. Care is essential for building a more just and free society. He also suggests that caring for the environment is an extension of caring for others.

Adela Cortina (2013) advocates an ethic of care that emphasizes interpersonal relationships, human vulnerability, and social responsibility. Her critical approach to individualism and her emphasis on social justice provide a comprehensive perspective on how care should be a fundamental value in contemporary ethics.

Bernardo Toro (2018), a Colombian educator and thinker, has approached the ethics of care from a critical and humanistic perspective. His approach focuses on the need to build a society based on respect, interdependence, and mutual responsibility, because caring implies recognizing the dignity of others and acting accordingly.

Torralba, 1998; Torralba, 2000; Torralba, 2005 explores the concept of care as an essential element of the human condition. From his anthropological perspective, care is not only an action but also a manifestation of our relational nature. Human beings, by their very essence, are beings who need to care and be cared for, which implies an intrinsic bond with others. Interaction and mutual care are aspects that shape our humanity. Vulnerability and need are essential characteristics of human beings, manifested in emotional and physical dependence from birth. This author emphasizes that caring implies a commitment to the other, recognizing their dignity and value. This commitment manifests itself in various ways: in the family, the community, and interpersonal relationships. Caring, therefore, is a practice that benefits the recipient and enriches the caregiver, establishing a circle of reciprocity. This idea recovers an essential aspect of the first meaning of accompaniment, which involves two people. It refers to those who are actively and committedly involved in the relationship, exposing themselves in an authentic and vulnerable way, and this applies to both the person accompanying and the person who is being accompanied. In both cases, they each reveal themselves as they are, accepting their own faults and committing themselves to the person with whom they are relating.

In all these theories from the last century, care is positioned as a practice that humanizes and strengthens communal life.

The COVID-19 pandemic highlighted the urgency of care, demonstrating the need for support for the elderly and the sick, and showing that this act strengthens community ties in times of crisis. However, the risk of turning care into a mechanical, depersonalized action requires us to reclaim its humanizing dimension, inseparable from accompaniment. At this point, it is worth remembering the meaning of accompaniment as a personal response, because in the face of this possible neglect, comprehensive accompaniment meets this need as a humanizing response. And this response is not theoretical, but personal and human: “The response is another life: that of the person who accompanies and sets out on the journey freely and committed to the destiny of the person being accompanied” (González Iglesias and la Calle Maldonado, 2020).

In the current context, technology can facilitate care but also create emotional distance. Human relationships remain fundamental to authentic and meaningful care, as “accompaniment is what makes suffering bearable, humanizing technology” (Hirsch, 1984). In this regard

The act of caring can and should benefit from technological tools, but, in itself, it is a human, and therefore personal, act, which means that the *physical* presence of the caregiver cannot be replaced under any circumstances. Humanising technology is a way of caring for the sick (Torralba, 1998).

Care is not restricted to the healthcare field, as already noted, but extends to education, parenting, and community life. It involves an ethical and relational commitment in which the dignity of the other is recognized and respected. As Domínguez Prieto (2022) points out, accompaniment is ultimately the art of caring and being cared for, including self-care as an expression of love for oneself and the value of accompanying others to care for them. This author states that:

Through accompaniment, we care for others in various ways: we encourage self-love, we strengthen them, we attend to their needs and encourage them to attend to their own, we promote the quality of their community relationships, and we inspire them to search for meaning in their lives (2022).

It is clear that these purposes transcend the educational sphere and can be realized in other areas, such as healthcare.

Care is a fundamental need that permeates all dimensions of human life. Fostering a culture of care is essential for individual and collective well-being. This call to action involves recognizing the importance of care and working to ensure everyone has access to the care they need. However, care is not only a necessity but also a moral imperative. The ethics of care is based on the idea that caring for others is a moral responsibility. By recognizing the shared vulnerability of the human condition, care becomes a manifestation of respect and dignity, in which human beings seek to alleviate others’ suffering and promote mutual well-being. This sense of ethical responsibility strengthens the bonds that unite us and gives rise to norms and principles that govern our interactions and promote community.

#### 2.3.1 The needs of the person being cared for

Once the anthropological assumptions about the person as a being in need of care have been addressed, some of the needs that any human being experiences at a time of particular vulnerability in their life are presented as a starting point for the care relationship:

- The need for love, to feel kind, worthy of being loved, to matter to others.
- The need for security, protection, respect, knowing that they will be provided with what they need, how they need it, in relation to basic needs (food and personal hygiene). There is an affective memory in every human being that is the experience of the womb, as a place of absolute security, which is awakened in this situation.
- The need to be relevant in the community in which one lives, so that the person feels that they are contributing, belonging, and making a difference in some way.
- The need to freely express emotions, ideas, and opinions.
- The need for autonomy to be respected and facilitated, not usurped.

Aware that the range of human needs from an anthropological perspective is much broader, those indicated here serve to remind us that in the process of care in any field, and especially in healthcare, which is our focus here, the needs experienced by human beings are alleviated, whether physical, psychological, social, or spiritual (Torralba, 1998).

### 2.4 The relationship of comprehensive care

Care is, above all, a relationship: it cannot be given without one. And it is probably the most qualified human relationship. There are two reasons for this statement: firstly, it can be said that it is the most qualified because its object is fragility, vulnerability, human weakness, that is to say, that which reveals humanity in all its greatness and preciousness. On the other hand, it can also be considered the most qualified because experience shows us that encountering the beauty of a vulnerable person brings out in the one who accompanies it a whole range of profound attitudes and virtues that spring from their heart and their vocation to care for vulnerability. This is a form of accompaniment that cannot be improvised but rather emerges from the depths of one’s being and requires more than skills and training; it requires work of the heart, which wisely enters into virtuous dynamism, to disarm oneself before the other and simply know how to be present before the mystery of suffering.

The person is a being who is and perceives himself as unitary in a multidimensional, bio-psycho-socio-spiritual way; that is why care for each human being must be integral. If care touches the being, it is constitutive of the person; it cannot but be integral, that is to say, it is given from the whole person of the caregiver to the whole person of the one being accompanied. Once again, something essential emerges in accompaniment: responding to anthropological needs by offering a personal response. In this sense, we must be wary of reductionism, which believes that attending to a person’s basic needs, such as eating or washing, is enough to care for them. In the caregiving relationship, forgetting that the person also needs their fears, concerns, desires, existential questions, and the impact of their care on their family or community to be addressed is a serious form of reductionism for the person being cared for. At this point, it is appropriate to pick up on the nuances pointed out by Verspieren regarding accompaniment in care:

The word “accompany” itself conveys an attitude, a conception of care, and a relationship with the sick person. Accompanying someone does not mean determining their path, showing them the way, imposing an itinerary on them, or even knowing the direction they will take; rather, it means walking alongside them, respecting their freedom to decide their path and the pace of their steps (1984).

We care for the whole person from our own wholeness. Hence, there is an intimate relationship between caring for others and caring for ourselves. We cannot care for a person in a way that is worthy of their dignity from a place of alienation or fragmentation. It is necessary to work on our own wholeness to holistically accompany and care for others.

Therefore, although the experience is and should be unitary, some aspects of the caring relationship are addressed below from various dimensions. This is done by considering some of the most relevant attitudes, virtues, and skills in the caring relationship. All of them are interdependent; none is sufficient on its own. They need each other to care for one another as they deserve. We have chosen to group them by type of need (physical, emotional, spiritual, and systemic) based on their impact on the experience of the person being accompanied.

#### 2.4.1 Care for physical needs

##### 2.4.1.1 Delicacy

Delicacy in treatment is often the gateway to a good caregiving relationship. Caregiving often brings us into the intimacy of the person we are caring for, a place we would not normally enter, and even less so with strangers. There is physical, emotional, and spiritual intimacy, and each requires a different tone that must be fine-tuned. Intimacy is that place where care for the person must be more delicate, more subtle, more careful. In intimacy, it is easy for us to be violated, invaded, robbed of what is most “ours,” because intimacy is close to “being.” Imagine we are moving around a room full of murano glass pieces. We will surely walk slowly and attentively, with healthy self-control, to respect each of these valuable pieces and not break them. This image clearly represents how we should move when we enter into a person’s intimacy.

Delicacy is a very elaborate expression of respect. According to the RAE, delicacy is “attention and exquisite consideration for people or things, in deeds or words” (Real Academia Española, n.d., definition 2). Delicacy is expressed with words, gestures, tone of voice, speed of speech, gaze, touch, and sometimes “without touching.” In general, delicacy is accompanied by calmness and serenity of spirit. It is not an artificial, rigid posture adopted at certain moments, but rather it emanates from the depths of the heart, where respect and love reside. It can be said that delicacy is one of the most qualified expressions of love, as it is the ability to grasp with finesse, perhaps intuitively, what love demands in each situation. Nor is it a quality that only some people have, but a virtue that, in a unique and personal way, everyone can and should cultivate.

Delicacy asks permission to “enter,” inquires subtly to discover the most appropriate way for that person, at that moment, and in the reality that must be “touched,” whether it be hygiene, tastes, personal needs.

##### 2.4.1.2 Respect for autonomy

Dependency is a challenge that must be faced sooner or later in life. There are needs that one was able to meet by oneself in the past, and which suddenly depend on others. In this situation, the person often does not choose the food they eat, the clothes they wear, the place where they go for a walk, or the times when this happens, because they depend on others and their possibilities. It should not be forgotten that the caregiver is also limited. The person being cared for may be hungry or listless, yet it may not be possible to meet their precise wishes. These are small examples, because the variety of ordinary life details, but they illustrate how important it is to know how to enter appropriately into the atmosphere of the dependent person.

The person being cared for must always feel that their autonomy is safe. It may be limited by circumstances, but never by the person who cares for and accompanies them. If the person experiences that respect, however limited they may be, they will be able to connect with their autonomy. In other words, a dependent person experiences the limits imposed by their illness or condition very differently from the limits imposed by *another person*. The former are experienced as a burden, but the latter are experienced as an injustice, an oppression, which generates greater suffering than the natural limitation itself, because the person feels that someone has taken away their freedom. caregivers can often confuse this, thinking that the natural limitations suffered by the person presuppose or justify the “usurpation” of their autonomy. Therefore, a good caregiver, who has internalized the value and dignity of the person, distinguishes between freedom of maneuver (which may be more or less limited) and autonomy, which is of a spiritual nature and therefore, in a way, is always possessed and never lost. Those who understand this become custodians, facilitators of others’ freedom.

In this regard, making important decisions for the life of the person being cared for deserves special mention. It is the ability to act with full knowledge of the facts, with sufficient information, and without pressure. In the final stage of life, respect for the dignity of the person and their autonomy takes on special importance. For this reason, the patient has the right to be informed and to participate in their care process.

#### 2.4.2 Caring for emotional needs

##### 2.4.2.1 Empathetic listening

Emotional needs are often overlooked in the caregiving relationship, yet these may be the needs that cause the most suffering if not adequately addressed. People need a welcoming, trusting environment where they can express feelings such as fear, uncertainty, and frustration, as well as their opinions and concerns. Listening is precisely the realm of trust and acceptance. It is therefore a place where the other person is welcomed, and their otherness is affirmed, to help them express their most genuine thoughts; that is why, in reality, listening precedes speaking (Escámez-Sánchez and Gil-Martínez, 2023). This requires emptying oneself of worries and inner ruminations in order to connect with the person we are caring for.

It is not just about listening to what the person says, but above all about detecting the emotional undercurrent of what they say. The empathetic caregiver perceives the sadness, pain, frustration, anger, joy, or enthusiasm of the heart, listening to the person’s heart and letting them know they are taking care of those emotions, that they are welcome and understood. In doing so, they help them process, accept, and realize what is happening to them, and even see more clearly so they can make a decision or change their attitude toward their vulnerability. In short, the fragile person is listened to so that they can speak and escape from the cell of loneliness in which they are often imprisoned. That is why listening is liberating and healing. Furthermore, listening is underpinned by another virtue that sustains it, which is patience.

##### 2.4.2.2 Confidentiality

Closely linked to empathic listening is confidentiality. The vulnerable person says many things to the person who accompanies them when they are immersed in situations of great suffering, deprivation, and limitations. They need someone trustworthy to share what is happening to them; they need a confidant. Often, a lack of understanding in the person’s immediate environment means they do not dare to communicate what is happening to them, what they need, or what worries them, for fear of judgment, rejection, shame, or the worry that they might cause suffering to others. Confidentiality is also a virtue, whereby the person who accompanies has the maturity to keep the secrets entrusted to them to themselves and remain silent. It is the intimacy of the other person that is entrusted to them, to be kept with respect and silence. That is why empathetic listening and confidentiality go hand in hand.

##### 2.4.2.3 Tenderness

It is essential that this listening be based on tenderness, another revitalizing attitude in the caregiving relationship. Tenderness is said to be the virtue of the strong, of those who are not afraid of evil, harshness, or violence, but who are able to enter and stand by others in difficult situations; it is a very powerful and “effective” expression of love. Tenderness disarms, breaks down barriers, fears, prejudices, and mistrust in the person being accompanied, and is the expression that “convinces” them that the caregiver truly has their best interests at heart. It is then that tenderness comforts and alleviates pain and suffering, removes fear, and gives confidence, security, and strength to face difficulties. In particular, tenderness is often conveyed through appropriate gestures, such as caresses, holding hands, or placing a hand on the shoulder or back. Gestures that communicate warmth, gentleness, closeness, affection, appreciation, and deep respect. This requires emotional maturity, inner freedom, and the integration of one’s own emotions on the part of the person who accompanies. “Tenderness is strength, a sign of maturity and inner vigor, and springs from a free heart, capable of giving and receiving love” (Rocchetta, 2016).

#### 2.4.3 Spiritual care

The person is a being of meaning, called to transcend themselves, to unity and fulfillment. For this reason, they are always accompanied by a certain vital dissatisfaction, because being contingent and incomplete, they have within themselves these longings for fulfillment and unity (Domínguez Prieto, 2011). Caring is not only about alleviating the tangible or material needs of the person, but also those intangible needs, those of the soul. Vulnerability sharpens and perhaps makes more painful the need for answers to the great questions harbored in the human spirit, and, at the same time, it is also an opportunity, because it predisposes and opens us to transcendence, to looking beyond and opening ourselves to the absolute.

In recent years, medicine has also addressed the spiritual accompaniment of suffering, presenting it as:

A deterioration in the ability to experience and integrate the meaning and purpose of life through connection with the self, others, art, music, literature, nature, and/or a power greater than the self, and refers to spirituality as the integration of the meaning and purpose of life (Benito et al., 2016).

For this reason, spiritual suffering leads to fragmentation, meaninglessness, emptiness, and despair. Below are some fundamental attitudes required to accompany this essential dimension of the person.

##### 2.4.3.1 Compassion

According to Torralba, compassion is the primary virtue necessary for care: “It is difficult to develop the action of caring without the experience of compassion, although the experience of compassion is not sufficient for the optimal development of care. It is a necessary but not sufficient condition” (Torralba, 2000). This author explains that “feeling compassion for someone is a habit of the heart that requires an ecstatic movement, a stepping *outside of oneself*, in order to understand the other in their context and take their pain into one’s own heart” (2000, p.137). This stepping outside of oneself and this taking on make compassion a virtue, a spiritual work of the caregiver’s heart. Compassion cannot be trained; it is suffered. It involves a personal cost, which is freely assumed out of love for the person who is suffering. In this sense, compassion takes charge of the depth of the person’s suffering, that which goes beyond other, more peripheral needs, and therefore brings deep hope, because it is capable of taking on meaninglessness, emptiness, and hopelessness.

However, to be such, it must be put into action; after the first movement of experiencing the suffering of others as one’s own, the person mobilizes creatively to help the other who is suffering. Action is the proof that the dynamism of compassion has been mobilized in the person.

Compassion requires that the person be *attentive,* that is, with an almost “contemplative” capacity to “receive” reality, to allow oneself to be affected by it, without violating it with what reality brings. This capacity, lived in this way, has enormous transformative power.

##### 2.4.3.2 Hospitality

The caregiver’s hospitality creates a presence that makes the person feel welcome. Based on Henry Nouwen’s idea in *The Wounded Healer*, one can truly welcome another as a caregiver when one recognizes one’s own vulnerability and wounds and turns them into a starting point for accompaniment. This communicates trust and authenticity to the person being accompanied. Let us not forget that “the person who accompanies does not have to be perfect but must be a witness of what they say” (González Iglesias, 2023). The caregivers need to feel at ease with themselves (Nouwen, 1998) because in order to “host” someone, one must feel comfortable in one’s own home, without fear, at peace, basically reconciled with one’s life. As a result, they will be able to create a “place,” a welcoming living space where the person being cared for feels free, confident, safe, where they can heal and “rest” from their burdens (Benito et al., 2016).

##### 2.4.3.3 Full presence

Full presence is essential in the caregiving relationship. As Barbero states:

When we are in the midst of suffering, we are jointly responsible not only for eliminating its causes, but also for our attitude toward it. And, ultimately, it will not be so much a problem that requires explanation, *but rather a mystery that demands presence* (2002).

Indeed, the nature of spiritual suffering is “mysterious” and requires knowing how to be present, letting go, and renouncing all easy remedies: consolation, advice, solutions. Those who accompany others are almost touching the “being” of the person and can only “be” and offer their good presence, which can reflect their own greatness to the person they are accompanying through a profound gaze (González Iglesias and la Calle Maldonado, 2020). A gaze that communicates to him or her that they are “more” than their illness or limitation, that they can transcend it, and helps them look beyond, so that the person themselves can also transcend. But they need someone who knows how to look at them in this way. It is a matter of “offering our presence in its fullness and, from there, empowering them to discover themselves connected to their own depth” (Benito et al., 2016).

#### 2.4.4 Care of the system

Vulnerable individuals live in a system of relationships that must also be addressed if comprehensive care is to be provided. Here, we will refer to the most common system: the family. Knowing how to accompany the family will be essential for the sick person.

The family—especially for those requiring regular care—is the fundamental pillar of care provision. In order to accompany the family, it is necessary to first understand the family dynamics, that is, the set of relational, structural, social, economic, and other elements that constitute the environment of the person being cared for. It is also necessary to accompany the family holistically, as a unit with the person being cared for.

Below are some elements of this family dynamic that must be understood in order to provide support. We may know the person because we care for them, but it is also essential to understand them within their network of relationships (Vázquez Castro et al., 2001):

- The quality and nature of relationships, along with cohabitation and communication styles: it is essential to understand these in order to identify whether there are relationships characterized by overprotection, lack of commitment, or what is known as a “conspiracy of silence” with little or poor communication, where information of interest to the person is often concealed out of fear, false compassion or not knowing how to communicate.
- Family dynamics: on a day-to-day basis, there may be people regularly present in the home, or everyone may work, and the person spends many hours alone.
- Family structure and stage of life: it is important to know who they live with, their family relationships, and their stage of life, as the situation changes depending on whether there are grandparents, young children, a single caregiver, teenage children, etc.
- Economic and social situation: the conditions of the home and the economic situation, whether solvent or not, are not irrelevant.
- The adjustments that the family has had to make in caring for the person, and the emotional cost that this may have caused. Understanding these circumstances helps to assess the flexibility and degree of unity among family members.

The system’s accompaniment seeks to harmonize the entire support network in the most appropriate and personalized way for the person being cared for.

In addition, it is necessary to identify the roles: who is the primary caregiver, and what roles the different family members adopt. Also, what are the relationships between caregivers, which can be complex? We must help them communicate, freely express how each one experiences the illness or the care situation, and how they want to be involved. It is important that everyone participates in some way, avoiding divisions, comparisons, or blame, which, when they occur, do significant damage to the family dynamic and to the person being cared for, who often feels like a burden or a “problem.” Verspieren states that:

Accompaniment consists of providing discreet help at each stage of development, so that the person never abandons themselves to loneliness or sinks into despair but is able to overcome their painful experiences (1984).

According to Torralba, discretion is a fundamental value because:

Caring for someone consists of providing *discreet help,* that is, help that is practically unnoticeable or that the recipient of such action hardly notices. When the act of caring is noisy, it loses its effectiveness and value, as the recipient becomes aware of the weight of such action and the difficulties it creates in their immediate environment. In the act of caring, the how, that is, the manner in which it is done, is fundamental, because even if the intention is good, if it is not articulated properly, then that action can be completely negative (Torralba, 1998).

The primary caregiver is usually someone with more time available and also a certain natural disposition toward caregiving. This is important because, without an inner disposition, it is common for the person to give up due to the stress of the role (Vázquez Castro et al., 2001).

Family system care seeks above all to facilitate communication among family members, fostering unity and coordination of efforts. Caring for a family member often generates considerable family conflict that must be managed, facilitating the expression of the emotional burden of care, unexpressed fears, concerns, inability to collaborate, and resistance to care. It is essential to foster a culture of openness and freedom in which all members can work together to develop a care plan.

On the other hand, it is also essential to support and sustain the primary caregiver. If the primary caregiver gives up, it can lead to a major family crisis that must be avoided, in which the person being cared for suffers most, as they need harmony and security among their caregivers. The caregiver is someone who usually emerges spontaneously within the family circle due to their availability or qualities. They are the person of reference for the person being cared for and the one who bears the entire physical, emotional, and moral burden of the person’s situation. In a way, depending on the degree of dependence, *they live the person’s life* in many respects, which can involve significant emotional strain, and the caregiver must be made aware of this. It is common for the person providing care not to realize this, precisely because they gradually become part of the cared-for person’s environment as if it were *their own*. They live their life and the other person’s life on many levels, which creates some imbalance. For this reason, it is very important to help the primary caregiver find ways to achieve their own balance.

### 2.5 Meaning and scope of accompaniment as culture and care

Having reviewed the anthropological assumptions, as well as the characteristics and attitudes of accompaniment and the comprehensive care relationship, it is necessary to consider the real meaning and scope of this approach in contemporary reality.

Today’s society, with its rapid technological change and globalization, poses an unprecedented challenge in all areas of human development. As a result of the so-called fourth industrial revolution, the social fabric has become increasingly complex and sophisticated, to the point of changing how we live, work, and relate to one another. The labor market requires professionals trained to build interpersonal relationships in this context of high complexity, change, and uncertainty.

At the same time, we live with a wide variety of psychological, social, and health problems typical of developed societies—harassment, anxiety, depression, adjustment and eating disorders, gender violence, sexual abuse, substance use and abuse, and addiction to new technologies, among others—which pose an enormous challenge for individuals, families, and communities. In the last 5 years, since the COVID-19 pandemic, many of these problems have been highlighted. Given these circumstances, it is worth asking ourselves what means are available to care for people in the sense and in the ways explained in this paper.

It is a fact that we are spectators—and perhaps protagonists—of an unparalleled contrast: despite living immersed in a dynamic of performance that promotes almost omnipotent individuals who stand out for their productivity, strength, and resilience, we too often find ourselves in need of rest, care, and security, which are inherent to our human condition. The lament of *burnout* syndrome is heard amid the society of fatigue (Han, 2012), which believes that “nothing is impossible.”

In this context, human beings need, more than ever, safe environments where they can be themselves and show their vulnerability. The culture of accompaniment can foster these environments, by taking the form of observable attitudes, values, and behaviors. In fact, the specific way of understanding the person and the relationships presented in this work reflects a specific Culture of Accompaniment, within which we affirm that people are at the center of our gaze and that meaningful encounters and relationships shape it. We are talking about a culture that humanizes, that enables development, growth, and living life to the fullest today. It implies that accompaniment is part of our DNA and must permeate or be taken into account in everything we do, in how we organize ourselves, and in how we are (González Iglesias, 2023).

This being the main meaning of accompaniment, it is possible to recognize the following implications in the culture that sustains it:

- It puts the person at the center, both their vulnerable condition and their well-being and development.
- It recognizes the need for healthy environments and promotes them, not only in certain individual or relational spheres but also at the structural and systemic levels.
- It reinforces the value of the community sphere and its action.

However, these implications must begin in the individual sphere and extend beyond it to achieve a social effect. As Sastre (2018) states:

For the change observed in individuals, one by one, to become a social reality, it must affect a relatively large portion of society. And even this, although necessary, is not sufficient. The change in question must become established (2018).

#### 2.5.1 Accompaniment for prevention and health promotion

In the health sector, there is a framework of standards established by the World Health Organization (WHO) that connects with the scope of this culture of support. Specifically, the 1986 Ottawa Charter for Health Promotion addresses this purpose.

The international consensus statement from the First International Conference on Health Promotion, held in Ottawa, Canada, in November 1986, includes, among its conclusions, several lines of action that point to the aforementioned implications for the health and comprehensive care sectors (WHO, 2021).

In Ottawa, the WHO defined health promotion as “the process of enabling people to increase control over their health in order to improve it” (WHO, 2021). It also proposed developing a health-friendly policy through five strategic actions.

Below are three of the five proposals presented by the WHO for promoting public health, demonstrating the fundamental coherence between these lines of action and the guidelines for a culture of accompaniment as the basis for comprehensive care in the health sector.

##### 2.5.1.1 Strengthening community action

Improving social action in health leads to improvements in collective health, and informal health care at the community level provides positive reinforcement for individuals and their peer communities. These are “informal groups or formalized associations that deal with specific problems” (López Fernández, 2014) as well as “associations aimed at non-health purposes that generate actions that have an impact on health” (López Fernández, 2014).

As previously stated, human beings are essentially relational beings. Therefore, fulfilling the need to belong to a community and benefiting from the value of participating in it are key aspects of growing alongside others, highlighting the opportunity to give to others and receive their support and understanding.

##### 2.5.1.2 Developing personal skills

Self-care is a direct and effective way to promote and improve health, given the impact of lifestyles on certain pathological habits:

Lifestyle and environmental factors often intersect, so changes in behavior must be accompanied by legislative measures or the provision of resources that enable them (López Fernández, 2014).

In this sense, individual and community support processes are an effective resource, promoting personal knowledge and, from this, acceptance and improvement. It is a process that promotes personal growth and development, often linked to changes in attitude and behavior toward healthier lifestyles.

##### 2.5.1.3 Creating healthy environments

Healthy environments are a field of health promotion that facilitate healthy choices for people. “The importance of the living or learning environment as a determinant of health is part of all social perspectives on public health,” including not only the psychophysical environment, but “also the social dimension, such as social networks and the legislative and cultural “environment” in which the lives of populations unfold” (López Fernández, 2014). Creating healthy environments, therefore, means preventing situations of vulnerability.

In practice, creating this “culture” and healthy environments involves developing support processes that promote real transformation in communities and institutions. As González Iglesias states:

Accompaniment is necessary, essential for change to really happen, not just intentionally: it must be grounded in a relational style with which all its members identify, in coherent practices that embody it, and with a structure that makes such a culture viable and sustainable. And this must occur at three interrelated or interdependent levels: individuals, teams, and organization (2023, p.41).

On the other hand, and beyond actions aimed at promoting health, it is worth noting here a second aspect inherent to the practice of support: its possible preventive nature. As the WHO points out, disease prevention “describes measures to reduce risks, prevent the onset of disease, halt its progress and reduce its consequences once it has taken hold” (2021, p.10). To establish a hierarchy of priorities, the WHO describes three levels of prevention. The primary level is aimed at reducing the prevalence of risk factors common to a range of diseases; the secondary level is aimed at the early detection of existing disease, in order to halt or delay its progression and effects; and the third level refers to strategies to prevent or reduce the risk of deterioration or complications from disease. The field of prevention is oriented toward people who are ill or at risk of becoming ill, unlike health promotion, which targets the healthy population. At each of these levels, and to the extent that accompaniment reduces risks, prevents disease, or slows its progress, and promotes the creation of healthy environments, its preventive nature is also evident.

If this is the case, then the people involved in the accompaniment relationship and in the network of relationships that generate this environment are called upon to perform an essential function for society, not only in health promotion but also in preventive care.

Finally, this classification enables identification of profiles likely to participate in different accompaniment initiatives: patients, primary caregiver, family members, healthcare professionals, etc. A thorough analysis would enable gauging the scope of each proposal, recognizing the specific value of support as preventive care or its contribution to health promotion.

## 3 Discussion

The reflection developed throughout this work allows us to recognize that care, far from being a mere assistance practice or a response to fragility, constitutes a fundamental anthropological category that defines the human condition. Insofar as human beings are relational and vulnerable, care reveals itself as the fullest form of relationship, in which responsibility, compassion, and recognition of the dignity of the other converge. This approach distances itself from utilitarian conceptions of care, emphasizing its ontological nature and its transformative potential for both the person being cared for and the person accompanying them.

In this sense, the theoretical results presented coincide with the postulates of authors such as Torralba (2000), Torralba (2005), and Verspieren (1984), showing that accompaniment is not reduced to a set of techniques or skills, but is, above all, an attitude of integral presence that encompasses the bio-psycho-socio-spiritual dimensions of the person. The caring relationship thus becomes a privileged space for encounter, where vulnerability is not perceived as a deficiency, but as an opportunity for mutual growth.

Likewise, the discussion allows us to verify that the practice of accompaniment is deeply consistent with the lines of action established by the World Health Organization (WHO) in the Ottawa Charter (1986) for health promotion. Indeed, by creating healthy environments, strengthening community action, and developing personal skills, accompaniment is an effective means of health promotion and prevention, offering a comprehensive perspective that transcends the limits of traditional clinical care. This convergence confirms the hypothesis and establishes the relevance of accompaniment as a cross-cutting strategy for public health and human development.

On the other hand, the analysis carried out shows that comprehensive care can only be sustained if there is a culture of accompaniment that enables it (González Iglesias, 2023). Such a culture implies a paradigm shift in contemporary relational models, often characterized by productivity, fragmentation, and social acceleration (Han, 2012). In contrast to this “performance society,” accompaniment proposes a return to the centrality of the person, to the value of presence, and to the recognition of human interdependence as a source of meaning and health.

A relevant aspect emerging from this reflection is the formative and preventive dimension of accompaniment. Formative, because the caregiver needs continuous inner work that allows them to care from their own integrity; preventive, because the establishment of relational environments based on empathy, tenderness and hospitality reduces the incidence of avoidable processes of suffering—such as isolation, depersonalization or emotional exhaustion—both in the person being cared for and in the caregiver themselves. In this context, self-care is an essential condition for truly comprehensive care.

Finally, it should be noted that accompaniment, conceived as a relational style and social practice, transcends the healthcare sphere to become a cultural and ethical proposal. It promotes a way of inhabiting vulnerability through reciprocity and hope, where suffering becomes a place of encounter rather than exclusion. This is where it’s humanizing potential lies: in making it possible for human beings, even in their fragility, to find paths to fulfillment, meaning, and shared transcendence.

Following Nouwen (1998) philosophy of the Wounded Healer, this accompaniment requires the person accompanying to live humility, authenticity, and reversibility in the relationship 100%.

It can be said that chronic pain and suffering bring a person face-to-face with reality in all its harshness. That is why they need and deserve an open-hearted relationship with the person accompanying them, who must be willing to enter that space without fear, without answers, on tiptoe, in silence, and with the strength of a compassionate heart. This seems to be the most appropriate way to walk the path of possible fulfillment that comprehensive care offers both parties across all areas, especially in healthcare.

The analysis developed allows us to affirm that accompaniment, understood through an anthropology of care, constitutes a coherent and necessary response to the contemporary challenges of healthcare. In contrast to care models focused predominantly on illness, technical efficiency, or the standardization of processes, accompaniment introduces a relational logic that places the vulnerable person at the center of care practice.

The anthropology of care proposed by Torralba offers a solid foundation for this understanding, conceiving care as a constitutive dimension of the human being rather than an accessory or merely instrumental activity. From this perspective, accompanying does not mean directing, replacing or deciding for the other, but rather being with the person in their life process, recognizing their dignity, uniqueness and vulnerability. Comprehensive accompaniment thus emerges as a praxis that responds to an ontological need of human beings: the need to be cared for in order to develop and sustain themselves in situations of fragility.

Recent international literature on person-centered healthcare reinforces this orientation. The shift toward *personalization* and *person-centered* care reflects a profound cultural change that recognizes the person as an active subject of care rather than merely a recipient of technical interventions (McCormack and McCance, 2021; Tyagi et al., 2025; Winance and Bertrand, 2024). In this context, accompaniment takes on a longitudinal and relational character, transcending the specific clinical act and focusing on the comprehensive support of the person throughout the health-illness process.

Likewise, reflection on vulnerability allows us to understand that humanized care does not consist solely of mitigating deficits, but rather in responding ethically to the constitutive fragility of the human condition. Recognizing the ontological, situational, relational and technological dimensions of vulnerability broadens our understanding of care and highlights the need for accompaniment practices that integrate the human dimension in the face of the risks of depersonalization associated with the technification of healthcare.

In this sense, comprehensive accompaniment is a key mediator for the humanization of care, articulating presence, compassion, respect for autonomy, and attention to the emotional and spiritual dimensions of the person. It is not an alternative to technical competence, but rather its necessary integration into a truly person-centered care practice.

Overall, the analysis developed allows us to affirm that accompaniment constitutes an ethical and relational response to the contemporary challenges of healthcare, especially in contexts of increasing technological and care complexity. This article contributes to current debates on the humanization of healthcare by conceptualizing accompaniment as a relational and ethical framework that integrates vulnerability, person-centered care, and the anthropological foundations of comprehensive care.

## Data Availability

The original contributions presented in the study are included in the article/supplementary material, further inquiries can be directed to the corresponding author.
